# Resting position of the head and malocclusion in a group of patients with cerebral palsy

**DOI:** 10.4317/jced.51129

**Published:** 2014-02-01

**Authors:** Victoria Martinez-Mihi, Francisco J. Silvestre, Lorena M. Orellana, Javier Silvestre-Rangil

**Affiliations:** 1 Associate Professor, Department of Stomatology, University of Valencia, Valencia, Spain; 2 Assistant Professor, Department of Stomatology, University of Valencia, Valencia, Spain; 3 Dentist of the Red Cross Clinic for Special Patients, Valencia, Spain

## Abstract

Cerebral palsy are found as a result of these disorders, along with associated neuromuscular functional alterations that affect the resting position of the head. In this context, the resting position of the head could be responsible for several skeletal and dental occlusal disorders among patients with cerebral palsy. Objective: To assess the presence of malocclusions in patients with cerebral palsy, define the most frequent types of malocclusions, and evaluate how the resting position of the head may be implicated in the development of such malocclusions. Study design: Forty-four patients aged between 12-55 years (18 males and 26 females) were studied. Occlusal conditions, the Dental Aesthetic Index (DAI), changes in the resting position of the head, and breathing and swallowing functions were assessed. Results: Orthodontic treatment was required by 70.8% of the patients, the most frequent malocclusions being molar class II, open bite and high overjet. These individuals showed altered breathing and swallowing functions, as well as habit and postural disorders. The resting position of the head, especially the hyperextended presentation, was significantly correlated to high DAI scores. Conclusions: The results obtained suggest that patients with cerebral palsy are more susceptible to present malocclusions, particularly molar class II malocclusion, increased open bite, and high overjet. Such alterations in turn are more common in patients with a hyperextended position of the head.

** Key words:**Cerebral palsy, malocclusion, head position, disabled patients.

## Introduction

Cerebral palsy (CP) is characterized by a series of movement and postural development disorders that cause activity restrictions, and which are attributed to non-progressive alterations in the immature brain of the fetus or young child. The motor disorders often manifest together with sensory, cognitive, communicative, perceptive and behaviour alterations, and/or epileptic crisis. The estimated prevalence is 2-3 cases per 1000 live births in the western world (1-3).

Cerebral palsy can be classified according to the phase in which it develops (prenatal, perinatal or postnatal), or according to the etiopathogenic factors involved (genetic, hematological, immunologic, infectious, metabolic-biochemical, and brain maturation factors)(3,4).

Five types of CP can be distinguished, based on the main clinical manifestations. The most frequent presentation, representing 50-70% of all cases, is spastic CP, characterized by hypertonic muscular spasticity, hyperreflexia, abnormal postures and difficulties performing voluntary movements. Depending on the number of affected limbs, three clinical variants can be distinguished: quadriplegia, hemiplegia and diplegia. Dystonic-athetotic CP in turn accounts for 15% of all cases, and is characterized by muscle tone fluctuating between hypotonia and hypertonia, with the appearance of involuntary movements, anomalous postures, and altered voluntary movements. Ataxic CP (5-10% of all cases) is characterized by hypotonia, balance alterations and uncoordinated voluntary movements. Hypotonic CP in turn is the most severe and less frequent type, and is the result of total brain involvement. Finally, we have the mixed forms, which are characterized by spastic phenomena of the four limbs and dyskinetic-athetotic manifestations of the trunk and head (5,6).

Malocclusions are among the most severe and frequent oral alterations found in patients with CP. The literature offers evidence both in favor and against the existence of an increased prevalence of malocclusions in such patients, compared with the normal population. In turn, among the different forms of malocclusion, molar class II, with increased open bite and overjet has been suggested to be the most frequent presentation. Likewise, consensus is lacking regarding which factors contribute most to malocclusion in patients with CP, though most authors point to the classical form-function relationship as the root cause. According to this hypothesis, functional orofacial neuromuscular alterations (particularly breathing and swallowing problems) are the key factor underlying the development of malocclusion (7-14). However, on examining the factors involved in the development of dental occlusion, we see that the resting position or postural position of the head exerts a decisive influence (15,16).

Some studies have demonstrated that forward resting positions present a greater craniofacial angle, which is associated with vertical growth patterns, long faces and posterior rotation of the mandibular axis - resulting in increased vertical mandibular growth and increased lower facial height. On the other hand, flexed resting positions are associated with horizontal growth patterns, short faces and anterior rotation of the mandibular axis - inducing horizontal mandibular growth, and a decreased anterior facial height (17).

The mechanism that best explains these associations is soft tissue stretching together with the existence of a muscle feedback system which would be responsible for maintaining the resting position of the head and neck (18). Any factor acting long enough to alter the resting position of the head would be responsible for soft tissue stretching in one sense or other, and would be the ultimate cause of patient occlusion / malocclusion.

In cases with the head located in a forward position or in hyperextension, the soft tissues would exert retrusion forces upon the facial bones. The cervical fascia would stretch the mandibular body downwards, favoring posterior rotation of the mandibular axis and inducing its vertical growth. The molars in turn would over-erupt to prevent the loss of occlusal contact, and the incisors would loose their interproximal contact, leading to open bite and use of the tongue to seal the oral cavity when swallowing, with interpositioning of the tongue between the incisors. On the other hand, the position of the hyoid bone would be altered, and hence the resting position of the tongue, which would lie in a lowered position, without touching the palate, thereby favoring a narrowed upper jaw (19,20).

The final position of the upper incisors would be determined by the action of the upper lip upon the basal and alveolar maxillary bone. A short and hypotonic lip exerts retrusion action upon the basal bone, stimulating protrusion of both the alveolar bone and the upper incisors (21,13). Thus, the resting position the head would be the mechanism best explaining an increased prevalence of malocclusions - class II being the most frequent presentation, with increased open bite and overjet.

The present study was designed to determine the most frequent type of malocclusion in a group of patients with CP, and to analyze the influence of the resting position of the head upon the development of malocclusion.

## Material and Methods

- Study sample

An observational cohort study was made of patients with CP. In order to obtain a representative sample, a random selection of individuals from different special educational centers in the region of Valencia (Spain) was carried out. The following inclusion criteria were established: an objective diagnosis of CP of any type, some degree of patient cooperation, a minimum age of 12-15 years (the age recommended by the World Health Organization (WHO) for examining permanent teeth), a Dental Aesthetic Index (DAI) of > 25 (22), and signed informed consent from the parents and/or tutors before performing the study. The exclusion criteria were: poor patient cooperation, missing upper or lower molars, and missing anterior teeth. The absence of a single incisor, or an incisor replaced by any type of prosthetic solution, was not regarded as an exclusion criterion, in the same way as the presence of concomitant diseases.

The study sample consisted of 44 patients (out of an original total of 48 subjects): 18 males (40.9%) and 26 females (59.1%), between 12-59 years of age. A total of 20.4% (n=9) had no mental retardation, 20.4% (n=9) had mild mental retardation, 18.2 (n=8) suffered moderate mental retardation, and 18.2% (n=8) and 22.7% (n=10) presented severe and deep conditions, respectively. Fifty percent of the sample had been diagnosed with epilepsy. Regarding the type of CP, the distribution was as follows: 24 subjects (70.4%) had spastic tetraparesis, 5 (11.4%) hemiparesis, and 2 (4.5%) hemiplegia. Two patients each presented hypotonic, ataxic and athetotic CP, while the remaining 7 subjects (15.9%) presented mixed forms.

- Clinical examination

In order to explore the subjects, number 5 intraoral mirrors and the WHO periodontal probes were used. A Glatzel mirror was used to evaluate breathing function, and glasses of water were used to assess swallowing. Explorations were made in adequate places in the different centers, and most of the patients were examined sitting on their own wheel chairs or in common plain chairs. The study data were recorded on specific forms.

- Study design

The presence or absence of control of the resting position of the head was clinically evaluated, as well as the type of control. We determined whether control followed the body axis or contrarily was performed in a hyperextended or a flexed position. Breathing function was specified as nasal, oral or both, while swallowing function was classified as normal or atypical. Data on occlusion, molar and canine class, overjet, vertical incisal relationship (measured in mm) were obtained, followed by recording of the DAI score (22), which allows clinicians to classify the severity of malocclusion on a numerical scale: ≤ 25 indicates minor malocclusion needing no treatment; 26-30 indicates definite malocclusions with elective treatment; 31-34 indicates severe malocclusion with strongly desirable treatment; and ≥ 35 indicates severe or incapacitating malocclusion in which treatment is considered mandatory.

- Statistical analysis

The data were entered in a MS Excel database and posteriorly analyzed with the SPSS version 18.0 statistical package. The nonparametric Kruskal-Wallis test was used for the comparison of two categorical quantitative variables with a non-normal distribution. The Mann-Whitney U-test in turn was used to compare qualitative variables with a non-normal distribution. Statistically significant results were considered for p≤0.05 and p≤0.01.

## Results

Based on the DAI score, the sample was distributed as follows: 8.3% (n=4) of the subjects presented a score of ≤ 25, corresponding to Level 1; 20.8% (n=10) presented a score of 26-30, corresponding to Level 2; 8.3% (n=4) presented a score of 31-34, corresponding to Level 3; and the remaining 62.5% (n=30) were assigned to the last level (DAI ≥ 35), where treatment is considered mandatory.

Data referred to head resting position, lip resting position and breathing are presented in  Table 1.

**Table 1:**
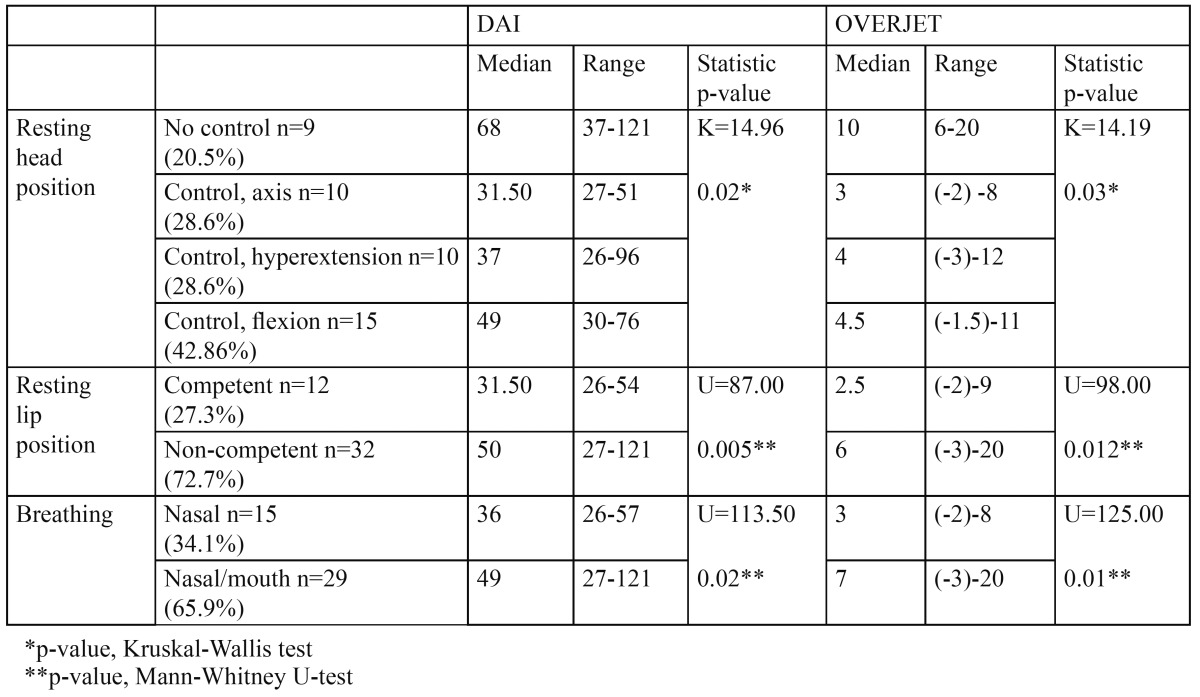
Statistical results of the association between DAI and overjet with resting head position, resting lip position and breathing.

Swallowing proved functional in 6.8% (n=3) of the patients, while tongue protrusion was seen in 20.4% (n=9) of the cases. In 59.1% (n=26) of the patients the tongue not only protruded but was also interposed between the incisors, and 13.6% (n=6) showed chin muscle contraction.

Regarding molar occlusion, 50% (n=22) of the cases corresponded to class II, 22.7% (n=10) to class I, and 27.3% (n=12) to class III. As refers to the canine relation, 70.5% (n=31) of the patients corresponded to class II, 15.9% (n=7) to class I, and 13.6% (n=6) to class III.

**Figure 1:**
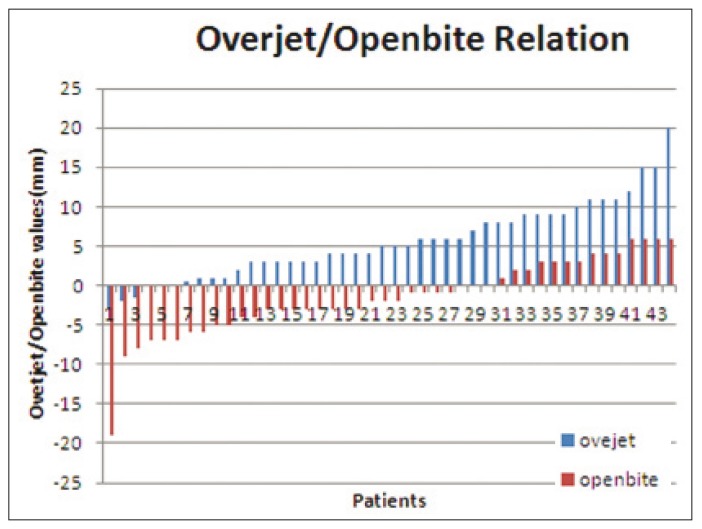
Data referred to overjet and open bite.

Data referred to overjet and open bite are shown in figure 1: 68.2% (n=30) of the patients had high open bite values, and 45.5% (n=20) showed important overjet.

The statistical results of the association between DAI and overjet with resting head position, resting lip position and breathing are shown in  Table 1.

The relationships between DAI and atypical swallowing and molar class II were significant (p<0.05).

Open bite was also found to be significantly associated (p<0.05) with atypical swallowing and with a resting position of the head in hyperextension.

No associations between overjet, molar class or anterior vertical relation and the type of cerebral palsy or the degree of mental retardation were found.

## Discussion

In the same way as in our series, Guerreiro (12) used the Dental Aesthetic Index (DAI) to study a group of 41 patients with cerebral palsy (CP). Seventy-five percent of the subjects with permanent teeth yielded DAI scores of over 35, corresponding to severe disabling malocclusion. Other studies have recorded a higher prevalence of malocclusions in patients with CP compared with the controls, though without specifying the evaluation method used (14). In contrast, other authors have found that only 35.4% of the CP group needed orthodontic treatment (23). This does not coincide without own study, in which only four subjects of the initial series of patients with CP (n = 48) had no malocclusions and were thus excluded from the study, while 91.6% required some type of orthodontic treatment - whether elective (20.83%), highly desirable (8.3%) or mandatory (62.5%). On comparing these data with the results of a study on the prevalence of malocclusions in children in the region of Valencia (Spain), based on the DAI, the distribution was found to be inverted, since only 16.1% of the children corresponded to the highly desirable and mandatory orthodontic treatment groups, while the great majority (83.8%) either required no treatment or only elective treatment (22).

In coincidence with some studies (24), we found molar class II to account for 40% of the sample, though other authors have reported prevalences as diverse as 75.85% and 26.4% (8,10). These discrepancies are in strong contrast to the increased overjet described by all authors among patients with CP versus the controls (8,10,13,14,24). In our study, increased overjet was seen in 45.5% of the cases, which is high compared with the normal population, where the reported prevalence is 8.5% (22). In the literature, the data referred to open bite vary considerably from 46.6% to 75.8%. In our series, 68.2% of the patients presented open bite, which is regarded as much higher than the prevalence found in the healthy population in the region of Valencia (0.6%)(23). Although not all the studies have recorded a statistically significant relationship between open bite and CP, there is general agreement that the values are higher among patients with CP than in the controls (8,23).

We recorded no statistically significant data relating a higher degree of malocclusion to mental retardation or any type of CP. Some authors have reported spastic cerebral palsy to be related to molar class II and increased open bite and overjet. Other investigators in turn have correlated increased mental retardation to more severe malocclusion, and specifically to increased overjet (8,11,14). Such variability among different studies may be due to the great diversity of clinical signs in patients with CP.

The mechanisms best able to explain the development of malocclusions are a change in resting position of the head, maintained by the muscle feedback system, and the soft tissue stretch hypothesis. At extraoral level, no studies in patients with CP have offered information on the postural position of the head. In our study, the resting position of the head was significantly correlated to high DAI values, and more specifically to the hyperextended position, both in patients who control the position of the head and those who do not (17,18).

Breathing function, especially nasal and mouth breathing, showed a statistically significant association to the resting position of the head. This factor, if maintained over time, could force modification of the resting position of the head and could lead to the development of an oral breather (17). In our series, the percentage of breathers who used both the nose and mouth was 65.9%, which is far higher than the percentages reported in other studies (ranging from 33.3% to as much as 42%)(8).

Another variable directly related to the resting position of the head is overjet, since it increases when hyperextended positions are evident. According to the tissue stretch theory and the neuromuscular feedback mechanism, the inclination of the upper incisors is the result of upper lip action upon those teeth. Among patients with CP, we observed incisor proclination as a result of lip action. Because of its hypotonicity, the lip is unable to seal the mouth, and consequently, it lays over the upper basal bone, where it acts retruding. The statistically significant relationship found in our study between increased overjet and a lack of lip competence confirms this theory (13,19,21).

Open bite also showed a statistically significant association to the resting position of the head. In this context, hyperextended positions were related to high open bite values. This can be explained by a change in the direction of growth, which occurs along the posterior mandibular axis when the head is hyperextended. Furthermore, it can be assumed that tongue interpositioning is not a cause of open bite, but a consequence of the latter (10,16,24).

The variables which are able to change the resting position of the head are oral breathing, hearing disorders, visual disorders, suture growing disruptions, changes in postures caused by muscular disorders, and habits. The consequences vary depending on genetic factors and the degree of functional adaptation of the structures.

These patients have a crucial need for orthodontic treatment, but the possibility of offering such treatment is limited. In this context, if it were possible to gain control or even modify the resting posture of the head from an early age, avoiding hyperextended patterns, malocclusions (or at least their severity) could be reduced as a result.

## Conclusions

Based on the DAI scores obtained, patients with PC are seen to have an important need for orthodontic treatment – molar class II, increased open bite and high overjet being the most common occlusal conditions.

The resting position of the head can be considered another determinant factor in the development of malocclusions in these patients, since neuromuscular alterations can lead to muscle changes and favor the development of malocclusions. In this context, patients with CP who present postural disorders would be more likely to develop morphologic disorders in the facial area, ultimately influencing the inferior facial third and favoring the development of malocclusion.

In our study, increased orthodontic treatment needs were associated to a hyperextended position of the head both in individuals who control the position of the head and in those who do not. A hyperextended resting position of the head can contribute to the development of class II malocclusions, increased overjet and greater open bite, which can be explained by the muscle feedback system and the tissue stretch theory.
